# Eating for performance: an evaluation of food consumption patterns and adherence to dietary guidelines in elite wheelchair basketball players

**DOI:** 10.7717/peerj.21715

**Published:** 2026-09-16

**Authors:** Aurora Norte, María Miana, David Romero-García, Isabel Sospedra, Eva Ausó, Ana Cifuentes, Sara Perpiñá-Martínez, Sara Aprea-Moreno, Pablo Leardy Palencia, Santiago Delgado Ortiz, José Miguel Martínez-Sanz, María Isabel Buceta Toro

**Affiliations:** 1 Applied Dietetics, Nutrition and Body Composition Research Group (DANUC), University of Alicante, Alicante, Spain; 2 Department of Nursing, Faculty of Health Sciences, University of Alicante, Alicante, Spain; 3 Faculty of Nursing and Physiotherapy ‘Salus Infirmorum’, Pontifical University of Salamanca, Madrid, Spain; 4 Optics, Pharmacology and Anatomy Department, Faculty of Sciences, University of Alicante, Alicante, Spain

**Keywords:** Wheelchair basketball, Dietary habits, Energy intake, Paralympic athletes, Nutrition assessment

## Abstract

This study evaluated dietary habits, hydration practices and adherence to nutritional recommendations in elite Spanish wheelchair basketball players, examining differences by sex and functional classification. Sixty-six athletes from first-division teams completed the validated Food4Me Food Frequency Questionnaire, and adherence was assessed using national dietary guidelines. Overall compliance with recommendations was low, the most athletes consumed fruits, vegetables, legumes, cereals and fish below recommended frequencies, while dairy, red meat and eggs generally met guidelines. Fruit intake was below recommendations in all women and in 91.2% of men, and a significant sex difference emerged only for dairy, with lower consumption in women. By functional classification, dairy intake was also lower among athletes with greater functional limitation. Most players (80.3%) did not follow any specific diet. Hydration practices were suboptimal, with 66.7% of women consuming ≤0.5 L of water during training compared with 15.8% of men, and athletes with grade 1 functionality drinking less water during matches than those with grade 4. These findings indicate limited adherence to dietary guidelines and insufficient hydration practices in this population, underscoring the need for tailored, disability-informed nutritional education strategies to support dietary adequacy and performance.

## Introduction

The Paralympic Games provide a unique context for athletes with permanent physical, visual, or intellectual impairments (Goosey-Tolfrey & Perret, 2025). These impairments require tailored approaches to training, performance, and nutrition according to individual needs. Among Paralympic disciplines, wheelchair basketball (WCB) is one of the most popular sports, with increasing international recognition (Ferreira da Silva et al., 2022).

WCB is a high-intensity intermittent team sport in which two teams of five players compete using wheelchairs, which are considered part of the player (Bloxham et al., 2001). All participants present impairments affecting the lower limbs to varying degrees (*e.g*., amputation, paralysis, or weakness) (Bloxham et al., 2001). The sport requires a combination of physical capacities such as speed, agility, strength, power, and endurance, as well as technical skills including propulsion, turning, dribbling, passing, and shooting (Gil et al., 2015). Game rules are largely similar to those of conventional basketball, with specific adaptations due to wheelchair use (International Wheelchair Basketball Federation (IWBF), 2025; BSR España, 2025). A key feature of this sport is the functional classification system, which classifies athletes according to their ability to perform specific game actions. Players are assigned a score ranging from 1.0 to 4.5 points, where lower scores indicate greater functional limitation (International Wheelchair Basketball Federation (IWBF), 2025; BSR España, 2025). During competition, the total score of the five players on court must not exceed 14 points (de Lira et al., 2010).

Nutrition is widely recognized as a key factor for optimal athletic performance (Broad, 2019; Thomas, Erdman & Burke, 2016). Nutritional requirements depend on factors such as training intensity, duration, and load, as well as individual goals, including body composition optimization for competition (Hertig-Godeschalk et al., 2023). In Paralympic sports, these requirements may be further influenced by disability-specific factors, such as functionality and clinical conditions, requiring individualized nutritional strategies (Broad, 2019; Scaramella, Kirihennedige & Broad, 2018).

However, there is still limited evidence regarding the nutritional needs and dietary intake of para-athletes, partly due to the heterogeneity of impairments (Goosey-Tolfrey & Perret, 2025). As a result, nutritional recommendations are often extrapolated from able-bodied athletes, despite known differences in energy expenditure and physiological demands (Scaramella, Kirihennedige & Broad, 2018; Figel et al., 2018; Islamoglu & Kenger, 2019; Flueck & Parnell, 2021; Ruettimann et al., 2021). Existing studies have mainly focused on energy and macronutrient intake, showing suboptimal dietary patterns characterized by high fat intake and insufficient consumption of carbohydrates, fiber, and legumes (Toti et al., 2022; Goosey-Tolfrey & Crosland, 2010; Deguchi et al., 2021; Raguzzini et al., 2021; Grams et al., 2016). Additionally, although dietary models such as the Mediterranean diet have been associated with improved performance and recovery (Peluso, Romanelli & Palmery, 2014; Pasquarella et al., 2019; Alonzo et al., 2019; Ciccotti et al., 2018), para-athletes often exhibit low overall diet quality, particularly in fruit, vegetable, and whole grain consumption (Joaquim, Juzwiak & Winckler, 2019).

**Figure 1:**
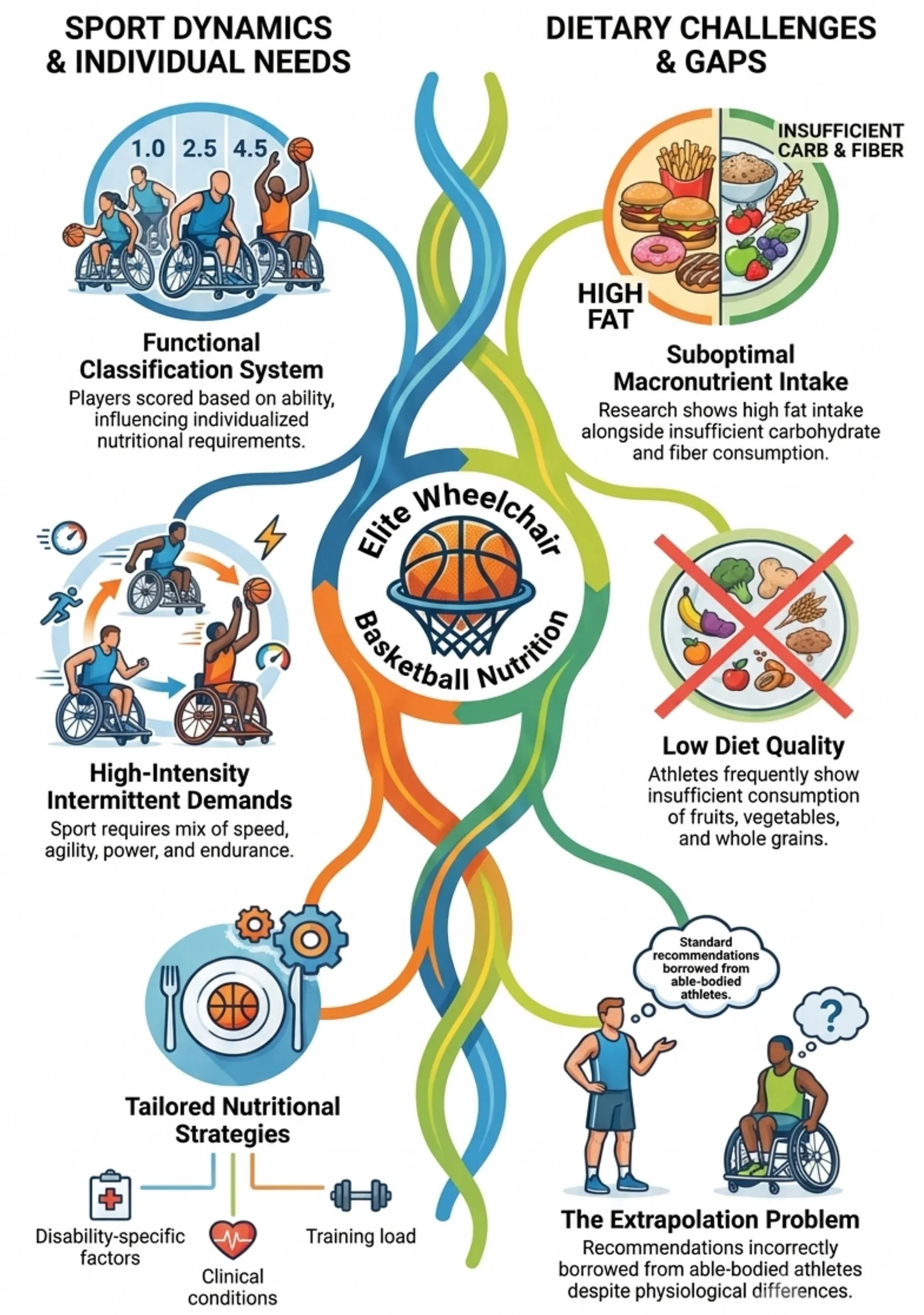
Summary of the diet of elite wheelchair basketball players.

Given these limitations, it is essential to better understand the dietary habits of athletes with impairments in order to develop tailored nutritional strategies (Fig. 1). To date, no studies have specifically examined dietary habits in Spanish first division WCB teams. Therefore, the aim of this study was to evaluate dietary habits, type of diet and motivations, hydration practices, and adherence to nutritional recommendations in elite WCB players, according to sex and functional classification. It was hypothesized that: (H1) dietary habits among elite WCB players would not meet the food frequency recommendations, showing a generalized low adherence; and (H2) most players would not follow a specific diet, while those who do would primarily do so for performance-related reasons.

## Materials and Methods

### Type of study

This is a descriptive, cross-sectional and non-experimental study (Martin et al., 2021) of the frequency of food consumption by Spanish elite WCB players. The study was prospectively registered in the ClinicalTrials.gov database (identifier: NCT07464899). In addition, the study design and manuscript development followed the STROBE-nut guidelines for nutritional epidemiology, an extension of the general STROBE recommendations for observational research (Lachat et al., 2016).

### Study population and settings

A convenience sampling method was used to recruit elite players from the Spanish WCB first division. Given the highly specific and limited nature of this population, the study aimed to achieve maximum representation of the top-tier competitive level. The final sample consisted of 66 players (57 men, nine women) from 10 of the 12 teams (83.3%) comprising the Spanish WCB first division. Considering the total number of federated athletes with physical disabilities in Spain (*n* ≈ 1,900 for the 2023–2024 season of the Spanish Federation of Sports for People with Physical Disabilities; FEDDF) (Federación Española de Deportes de Personas con Discapacidad Física, 2025), this sample size provides a 95% confidence level with a margin of error of 11.8%. While this margin is higher than in general population studies, it is considered acceptable and robust for descriptive research in highly specialized, hard-to-reach athletic cohorts. The inclusion of the vast majority of teams from the highest competitive category ensures the ecological validity and representativeness of the findings for elite WCB in Spain. It is worth noting that the total number of federation licenses covers all sports disciplines, age categories and competition levels of the federation. The inclusion criteria in the study were as follows: (a) belonging to a WCB team that competes at the national level; (b) being federated in wheelchair basketball; and (c) training a minimum of 3 days per week. The exclusion criteria for the study were: (a) being injured at the time of the evaluations and (b) having suffered an injury in the last month before the evaluations that prevented the anthropometric assessment from being adequately carried out. Table 1 describes the players’ data regarding age and training days of the participants, segmented by sex, as well as the chi-square test value and Cramer’s V, respectively.

**Table 1:** Descriptive data of the participants, according to sex.

Variables		Total (*n* = 66)		Sex						
				Female (*n* = 9)		Male (*n* = 57)		Statistical values		
		Md	IQR	Md	IQR	Md	IQR	$\chi^2$	*p*	V
**Age**		30	10	31	11	30	11			
**Years as a member of a sports federation**		10	3	10	6	10	3			
**Years at the national level**		10	3	9	7	10	3			
**Days of training**		5	1	5	2	5	1			
					**Rs**		**Rs**			
**Daily training time**	**1–1.5 h**	2 (3.0%)		1 (11.9%)	*1.5*	1 (1.8%)	*−1.5*	2.689	0.847	0.143
	**1.5–2 h**	15 (22.7%)		3 (33.3%)	*0.8*	12 (21.1%)	*−0.8*			
	**>2 h**	49 (74.2%)		5 (55.6%)	*−1.4*	44 (77.2%)	*1.4*			

**Note:**

$\chi^2$, chi-square test value; V, Cramer’s V; Rs: residual; Md, median; IQR, interquartile range. The italicized values represent standardized residuals or sub-category percentage distributions.

### Procedure

To select the sample, we contacted the FEDDF and the coaches of each of the participating teams. Once they agreed to participate, all players were informed of the objectives and method of the research, signed the informed consent before starting the research. After agreeing to participate, they received an email containing a link to the food frequency questionnaire (FFQ), which the WCB players could voluntarily, electronically, and anonymously complete through a Google Form. The questionnaire was active from February to April 2024, during the 2023/2024 competitive season.

### Instruments

This study utilized a questionnaire that had been previously used in similar studies (Sebastiá-Rico et al., 2023; Soto-Célix et al., 2021; Ventura-Comes et al., 2019). The selected questionnaire was validated for content, applicability, structure and presentation by University College Dublin and Crème Software Ltd., (Food4Me FFQ). It is a self-administered, online and semiquantitative FFQ (Fallaize et al., 2014), with its Spanish version prepared by Bejar LM and collaborators (Bejar, Sharp & García-Perea, 2016). It contains a four main sections. The first section collects the age, the team in which the subject plays (which identifies the subject’s sex) and functional category. The second section is focused on intake habits (frequency) of various food groups, water and alcoholic beverages during the last 28 days, such as: fruits, vegetables, legumes/pulses, tubers, pasta, rice, bread, whole grains, white meat, red meat, eggs, fish, nuts, fermented foods, soft drinks, commercial sweets, prepared/frozen foods, dairy products, plant-based beverages and alcohol (beer, wine, mixed drinks). Frequency of consumption was measured by selecting one of the following options: never or less than once a month, 1–3 times a month, once a week, 2–4 times a week, 5–6 times per week, once a day, 2–3 times per day, 5–6 times per day, and >6 times per day. Food portion size was estimated using photographs of a standardized portion, as well as an exact amount in grams or mL. Third section, consisting of three questions, collects information on players’ water intake habits throughout the day, during training and during matches. The last section has four questions designed *ad hoc* by the authors to collect information on whether the players follow any specific dietary pattern, their underlying motivations and who is the person/professional providing advice, if any. To ensure consistency in data collection, operational definitions were established for each dietary option based on current literature: (1) no specific diet: the participant does not intentionally restrict, modify, or track their food intake according to any structured nutritional philosophy or specific guidelines; (2) Mediterranean diet: a dietary pattern characterized by a high intake of minimally processed plant foods (fruits, vegetables, legumes, nuts, and seeds), olive oil as the primary source of fat, moderate consumption of fish and poultry, and low intake of red meat and sweets; (3) Paleo diet: a nutritional approach that mimics the presumed diet of Paleolithic humans, focusing on whole foods such as lean meats, fish, fruits, vegetables, nuts, and seeds, while strictly excluding dairy, grains, legumes, and highly processed foods; (4) flexible diet: a nutrition strategy focused on meeting daily macronutrient targets rather than restricting specific food sources, allowing for dietary flexibility in food selection; and (5) intermittent fasting: an eating pattern that cycles between voluntary periods of fasting and eating within a specific time window (*e.g*., the 16/8 protocol), without inherently dictating the types of food consumed (Mielgo-Ayuso et al., 2025).

Regarding its applicability to sports populations, although the questionnaire was originally designed for the general population, its structure covers the main food groups for all types of populations and essential for athletic performance, and it has been successfully used in numerous previous studies with athletes of different disciplines (Sebastiá-Rico et al., 2023; Soto-Célix et al., 2021; Ventura-Comes et al., 2019). This allows for a reliable qualitative assessment of dietary patterns in a sports context. To complement the FFQ and address the specific nutritional needs of athletes, data on sports-specific products (*e.g*., energy bars, gels, and sports drinks) were collected using an additional, specialized sports supplement frequency questionnaire. These results are not included in this report as they are being analyzed in a separate investigation to ensure a more specialized and detailed evaluation of sports supplements in this population.

### Assessment of compliance with dietary recommendations

To evaluate adherence to dietary frequency recommendations by sex and functional level, national dietary and sustainable nutrition guidelines for the Spanish population were used. Specifically, reference criteria were drawn from the Scientific Committee Report of the Spanish Agency for Food Safety and Nutrition (AESAN) on sustainable dietary and physical activity recommendations, complemented with official documents from the Spanish Ministry of Consumer Affairs (López García et al., 2022; Ministry of Social Rights, Consumer Affairs and Agenda 2030 of Spain, 2022). The reference frequencies were as follows: vegetables: 2–4 servings/day; fruits: 3–5 servings/day; cereals and derivatives: 4–6 servings/day; nuts: ≥3 times/week; dairy products: 2–4 servings/day; meat and meat products: 2–4 times/week; red meat: ≤2 times/week; fish and seafood: ≥2 times/week; eggs: 2–4 times/week; legumes: 2–4 times/week; water: 2–2.5 L/day. Each participant was classified as “meets”, “below”, or “above” the recommendation range for each food group.

Dietary diversity was assessed using the Dietary Diversity Score (DDS), which reflects the number of nutritionally relevant food groups consumed according to AESAN recommendations. The DDS was calculated for each participant as the sum of the food groups consumed. A group was assigned one point if it met the recommended frequency for that group (Kojima, Murayama & Suga, 2020; Ruel, 2003; Torheim et al., 2004). Ten food groups were included (water is not considered a food group in DDS), resulting in a possible score ranging from 0 to 10 points: fruit, vegetables, legumes, cereals, meat and meat products, red meat, eggs, fish, nuts and dairy products.

### Bias control

To minimize potential sources of bias, several methodological safeguards were implemented. The issue of selection bias was addressed by including all athletes from the participants competition. A comprehensive set of written instructions was provided with the objective of minimizing comprehension errors and the influence of social desirability bias. Recall bias, a potential limitation of self-reported instruments, was mitigated using concise and clearly worded items and by encouraging accurate responses. No data imputation was performed; only questionnaires that were complete and internally consistent were included in the analysis.

### Statistical analysis

The Kolmogorov–Smirnov test was applied to verify normality. As most variables were non-normally distributed, descriptive statistics were expressed as medians and interquartile ranges (IQR). For categorical variables—including sports-related characteristics, food group consumption frequencies, diet-related data, water intake patterns (daily, during training, and during matches), and compliance with dietary recommendations—Chi-square tests ( $\chi^2$) were conducted to detect differences by sex and functional classification. Standardized residuals and Cramer’s V coefficients were computed to assess association strength. For the analysis of the DDS, descriptive statistics were calculated. In addition, tertiles of DDS were calculated, and participants were assigned to each tertile to describe the distribution of dietary diversity in the sample. For sex, the Student’s t-test and Cohen’s d were used. The sample was divided into four groups, including functional scores 1 and 1.5 within group 1; functional scores 2 and 2.5 within group 2; functional scores 3 and 3.5 within group 3, and finally, functional scores 4 and 4.5 within group 4. The sample was stratified into these four groups (Supplemental Material) (International Wheelchair Basketball Federation (IWBF), 2025; BSR España, 2025). The significance level was set *a priori* at *p* < 0.05. Statistical analyses were performed using IBM SPSS Statistics v.25 (IBM Corp., Armonk, NY, USA).

### Ethical considerations

The study adhered to the ethical principles of the World Medical Association and the Declaration of Helsinki. Ethical approval was obtained from the Ethics Committee of the Pontifical University of Salamanca (Acta 15/12/2023) and the Ethics Committee of the University of Alicante (UA-2025-02-14). All participants received detailed information about the study and provided written informed consent prior to participation.

## Results

### Food consumption frequency

The frequencies of consumption across food groups for the total sample are presented in Table 2. Overall, players exhibited lower consumption frequencies than those recommended by national dietary guidelines in most food groups. For fruits and vegetables, the most frequently reported categories were 3–4 times per week (30.3% and 28.8%, respectively) and 1–2 times per day (28.8% and 27.3%), indicating intake below the recommended 3–5 daily servings. For specific vegetables, whole grains, nuts, and commercial sweets, the predominant frequencies were less than once per week (34.8%, 30.3%, 31.8%, and 30.3%) and 1–2 times per week (31.8%, 33.3%, 31.8%, and 33.3%). Regarding legumes, rice, fish, and dairy products, the most frequent category was 1–2 times per week (48.5%, 48.5%, 59.1%, and 25.8%, respectively). For tubers, pasta, bread, and red meat, the main frequencies were 1–2 times per week (39.4%, 30.3%, 24.2%, and 36.4%) and 3–4 times per week (39.4%, 42.4%, 25.8%, and 42.4%). White meat and eggs were primarily consumed 3–4 times per week (43.9% and 40.9%, respectively). Finally, fermented foods, soft drinks, processed/frozen foods, and plant-based beverages were most frequently consumed less than once per week (39.4%, 40.9%, 51.5%, and 69.7%, respectively).

**Table 2:** Frequency and confidence interval (%) of food group consumption in the total sample.

Item	<1 t/w	1–2 t/w	3–4 t/w	5–6 t/w	1–2 t/d	>3 t/d
Fruit	7.6 (1.2–14.0)	12.1 (4.2–20.0)	30.3 (19.2–41.4)	13.6 (5.3–21.9)	28.8 (17.9–39.7)	7.6 (1.2–14.0)
Vegetables	3.0 (−1.1 to 7.1)	18.2 (8.9–27.5)	28.8 (17.9–39.7)	19.7 (10.1–29.3)	27.3 (16.6–38.0)	3.0 (−1.1 to 7.1)
Specific vegetables	34.8 (23.3–46.3)	31.8 (20.6–43.0)	16.7 (7.7–25.7)	9.1 (2.2–16.0)	7.6 (1.2–14.0)	0.0 (0.0–0.0)
Legumes	21.2 (11.3–31.1)	48.5 (36.4–60.6)	21.2 (11.3–31.1)	6.1 (0.3–11.9)	3.0 (−1.1 to 7.1)	0.0 (0.0–0.0)
Tubers	9.1 (2.2–16.0)	39.4 (27.6–51.2)	39.4 (27.6–51.2)	10.6 (3.2–18.0)	1.5 (−1.4 to 4.4)	0.0 (0.0–0.0)
Pasta	9.1 (2.2–16.0)	30.3 (19.2–41.4)	42.4 (30.5–54.3)	13.6 (5.3–21.9)	4.5 (−0.5 to 9.5)	0.0 (0.0–0.0)
Rice	10.6 (3.2–18.0)	48.5 (36.4–60.6)	22.7 (12.6–32.8)	12.1 (4.2–20.0)	6.1 (0.3–11.9)	0.0 (0.0–0.0)
Bread	19.7 (10.1–29.3)	24.2 (13.9–34.5)	25.8 (15.2–36.4)	15.2 (6.5–23.9)	13.6 (5.3–21.9)	1.5 (−1.4 to 4.4)
Whole grains	30.3 (19.2–41.4)	33.3 (21.9–44.7)	16.7 (7.7–25.7)	15.2 (6.5–23.9)	4.5 (−0.5 to 9.5)	0.0 (0.0–0.0)
White meat	3.0 (−1.1 to 7.1)	19.7 (10.1–29.3)	43.9 (31.9–55.9)	27.3 (16.6–38.0)	6.1 (0.3–11.9)	0.0 (0.0–0.0)
Eggs	7.6 (1.2–14.0)	19.7 (10.1–29.3)	40.9 (29.0–52.8)	24.2 (13.9–34.5)	7.6 (1.2–14.0)	0.0 (0.0–0.0)
Fish	25.8 (15.2–36.4)	59.1 (47.2–71)	12.1 (4.2–20.0)	3.0 (−1.1 to 7.1)	0.0 (0.0–0.0)	0.0 (0.0–0.0)
Red meat	18.2 (8.9–27.5)	36.4 (24.8–48.0)	42.4 (30.5–54.3)	3.0 (−1.1 to 7.1)	0.0 (0.0–0.0)	0.0 (0.0–0.0)
Nuts	31.8 (20.6–43.0)	31.8 (20.6–43.0)	18.2 (8.9–27.5)	10.6 (3.2–18.0)	6.1 (0.3–11.9)	1.5 (−1.4 to 4.4)
Fermented foods	39.4 (27.6–51.2)	19.7 (10.1–29.3)	22.7 (12.6–32.8)	15.2 (6.5–23.9)	3.0 (−1.1 to 7.1)	0.0 (0.0–0.0)
Soft drinks	40.9 (29.0–52.8)	27.3 (16.6–38.0)	12.1 (4.2–20.0)	10.6 (3.2–18.0)	6.1 (0.3–11.9)	3.0 (−1.1 to 7.1)
Commercial sweets	30.3 (19.2–41.4)	33.3 (21.9–44.7)	27.3 (16.6–38.0)	6.1 (0.3–11.9)	1.5 (−1.4 to 4.4)	1.5 (−1.4 to 4.4)
Processed/frozen foods	51.5 (39.4–63.6)	36.4 (24.8–48.0)	7.6 (1.2–14.0)	4.5 (−0.5 to 9.5)	0.0 (0.0–0.0)	0.0 (0.0–0.0)
Dairy products	19.7 (10.1–29.3)	25.8 (15.2–36.4)	15.2 (6.5–23.9)	21.2 (11.3–31.1)	16.7 (7.7–25.7)	1.5 (−1.4 to 4.4)
Plant-based beverages	69.7 (58.6–80.8)	19.7 (10.1–29.3)	1.5 (−1.4 to 4.4)	3.0 (−1.1 to 7.1)	4.5 (−0.5 to 9.5)	1.5 (−1.4 to 4.4)

**Note:**

t/w, time/week; t/d, time/day.

Significant differences by sex were found only for the dairy products group (*p* < 0.001), see Table S1. The Table S2 presents the frequencies of consumption of the different food groups according to functional classification with statistically significant differences in the dairy products consumption (*p* = 0.022). As shown in Table 3, women reported a proportionally lower consumption, falling below recommendations, whereas men met the established intake levels in red meat, egg and dairy products. No significant differences were observed for other food groups (*p* = 0.120–0.842). No significant differences were observed in any of the dietary recommendations by functional classification (*p* = 0.109–0.705), see Table S4.

**Table 3:** Distribution (%) of dietary recommendation compliance by sex.

Recommendations	Sex								
	Female (*n* = 9)			Male (*n* = 57)			Statistical values		
	Meets	Below	Above	Meets	Below	Above	χ^2^	*p*	V
Fruit	0 (0.0%)	9 (100.0%)	-	5 (8.8%)	52 (91.2%)	-	0.854	0.355	0.114
*Residuals*	*−0.9*	*0.9*		*0.9*	*−0.9*				
Vegetables	0 (0.0%)	9 (100.0%)	-	2 (3.5%)	55 (96.5%)	-	0.326	0.568	0.070
*Residuals*	*−0.6*	*0.6*		*0.6*	*−0.6*				
Legumes	0 (0.0%)	8 (88.9%)	1 (11.1%)	14 (24.6%)	39 (68.4%)	4 (7.0%)	2.840	0.242	0.207
*Residuals*	*−1.7*	*1.3*	*0.4*	*1.7*	*−1.3*	*−0.4*			
Cereals	0 (0.0%)	9 (100.0%)	-	9 (15.8%)	48 (84.2%)	-	1.645	0.200	0.158
Residuals	−1.3	1.3		1.3	−1.3				
Meat and meat products	2 (22.2%)	1 (11.1%)	6 (66.7%)	8 (14.0%)	4 (7.0%)	45 (78.9%)	0.667	0.716	0.101
*Residuals*	*0.6*	*0.4*	*−0.8*	*−0.6*	*−0.4*	*0.8*			
Red meat	7 (77.8%)	-	2 (22.2%)	29 (50.9%)	-	28 (49.1%)	2.269	0.132	0.185
*Residuals*	*1.5*		*−1.5*	*−1.5*		*1.5*			
Eggs	2 (22.2%)	5 (55.6%)	2 (22.2%)	25 (43.9%)	13 (22.8%)	19 (33.3%)	4.248	0.120	0.254
*Residuals*	*−1.2*	*2.1*	*−0.7*	*1.2*	*−2.1*	*0.7*			
Fish	0 (0.0%)	9 (100.0%)	-	10 (17.5%)	47 (82.5%)	-	1.861	0.173	0.168
*Residuals*	*−1.4*	*1.4*		*1.4*	*−1.4*				
Nuts	2 (22.2%)	6 (66.7%)	1 (11.1%)	17 (29.8%)	42 (63.6%)	4 (7.0%)	0.343	0.842	0.072
*Residuals*	*−0.5*	*0.2*	*0.4*	*0.5*	*−0.2*	*−0.4*			
Dairy products	1 (11.1%)	8 (88.9%)	-	44 (77.2%)	13 (22.8%)	-	15.646	<0.001	0.487
*Residuals*	*−4.0*	*4.0*		*4.0*	*−4.0*				
Water	0 (0.0%)	9 (100.0%)	-	1 (1.8%)	56 (98.2%)	-	0.160	0.689	0.049
*Residuals*	*−0.4*	*0.4*		*0.4*	*−0.4*				

**Note:**

-, There is no recommendation; $\chi^2$, chi-square test value; V, Cramer’s V. The italicize values represent standardized residuals or sub-category percentage distributions.

Most athletes did not follow any specific diet (80.3%). Among those who did, the most common pattern was a flexible diet (10.6%), primarily motivated by health maintenance (12.1%). All athletes following a diet reported receiving guidance from a registered dietitian-nutritionist (19.7%). No significant differences were found in diet-related responses according to sex (*p* = 0.058–0.282) or functional classification (*p* = 0.319–0.921).

Table 4 summarizes water intake during the day, training, and competition. Significant sex differences were observed across all three contexts. During the day, the most commonly reported volume was 2.5 L (40.9%), with significant differences indicating lower intake among women (*p* = 0.002). During training, most players consumed 1 L (42.4%), with significant differences (*p* = 0.013), as a higher proportion of women reported ≤0.5 L. During competition, 1 L was again the most frequent amount (31.8%), and women exhibited lower intakes than men (*p* = 0.038). Table S5 shows the distribution of responses related to water consumption throughout the day, during training sessions, and during matches, according to functional classification.

**Table 4:** Distribution and confidence interval (%) of water consumption by sex.

Context	Response	Total (*n* = 66)	Women (*n* = 9)	Men (*n* = 57)	χ²	*p*	V
Daily water intake	0.5 L	3.0 (−1.1 to 7.1)	11.1 (−9.4 to 31.6)	1.8 (−1.7 to 5.3)	16.877	0.002	0.506
	1 L	15.2 (6.5–23.9)	33.3 (2.5–64.1)	12.3 (3.8–20.8)			
	1.5 L	13.6 (5.3–21.9)	44.4 (11.9–76.9)	8.8 (1.4–16.2)			
	2 L	27.3 (16.6–38.0)	0.0 (0.0–0.0)	31.6 (19.5–43.7)			
	2.5 L	40.9 (29.0–52.8)	11.1 (−9.4 to 31.6)	45.6 (32.7–58.5)			
During training	0.5 L	22.7 (12.6–32.8)	66.7 (35.9–97.5)	15.8 (6.3–25.3)	12.687	0.013	0.438
	1 L	42.4 (30.5–54.3)	33.3 (2.5–64.1)	43.9 (31.0–56.8)			
	1.5 L	22.7 (12.6–32.8)	0.0 (0.0–0.0)	26.3 (14.9–37.7)			
	2 L	10.6 (3.2–18.0)	0.0 (0.0–0.0)	12.3 (3.8–20.8)			
	None	1.5 (−1.4 to 4.4)	0.0 (0.0–0.0)	1.8 (−1.7 to 5.3)			
During matches	0.5 L	25.8 (15.2–36.4)	66.7 (35.9–97.5)	19.3 (9.1–29.5)	10.121	0.038	0.392
	1 L	31.8 (20.6–43.0)	22.2 (−5.0 to 49.4)	33.3 (21.1–45.5)			
	1.5 L	24.2 (13.9–34.5)	0.0 (0.0–0.0)	28.1 (16.4–39.8)			
	2 L	13.6 (5.3–21.9)	11.1 (−9.4 to 31.6)	14.0 (5.0–23.0)			
	2.5 L	4.5 (−0.5 to 9.5)	0.0 (0.0–0.0)	5.3 (−0.5 to 11.1)			

**Note:**

L, liters; $\chi^2$, chi-square test value; V, Cramer’s V.

Table 5 presents the DDS results for the study period. DDS values for the total sample ranged from 0 to 10. The median DDS was 2, with an interquartile range of 2. Overall, these results indicate the low level of dietary diversity present in the population during the study period.

**Table 5:** Dietary diversity score.

Variables	Total (*n* = 66)				Sex							
					Female (*n* = 9)				Male (*n* = 57)			
	Md	IQR	P25	P75	Md	IQR	P25	P75	Md	IQR	P25	P75
DDs points	2	2	1	3	1	2	1	2	2	2	1	3

**Note:**

DDS, Dietary diversity score; Md, median; IQR, interquartile range; P, percentile.

## Discussion

The present study analyzed food-consumption patterns and adherence to dietary recommendations (compliance with food consumption frequency) among Spanish WCB players. Our results should be interpreted in the Spanish context and taking into account the low representation of the female population. Overall, the findings indicate a suboptimal dietary profile, characterized by low adherence to recommendations for plant-based foods, carbohydrate-rich foods, fish, nuts, and water, together with excessive red meat consumption. Significant differences were limited to dairy intake according to sex, while most athletes did not follow a specific dietary plan. These results suggest that dietary quality in this population may be insufficient to fully support health, energy availability, and sports performance (Broad, 2019; Thomas, Erdman & Burke, 2016).

Previous studies in the Spanish general population have reported variable adherence to the Mediterranean diet, often showing suboptimal intake of key food groups such as fruits and vegetables, together with high consumption of meat and dairy products (García-Meseguer et al., 2014). Similarly, studies in Spanish athletic populations have described heterogeneous dietary patterns, characterized by insufficient intake of carbohydrate-rich foods and relatively high consumption of protein-rich foods (Muros et al., 2021). These comparisons should be interpreted with caution due to differences in dietary assessment methods. Nevertheless, the present findings suggest that WCB players exhibit dietary patterns that are not fully aligned with healthy dietary guidelines, particularly due to insufficient intake of plant-based foods and carbohydrate-rich sources.

Research on food-group consumption remains limited, specifically among athletes with physical impairments. Most previous studies have focused on total energy, macronutrient, or micronutrient intake rather than specific food groups (Toti et al., 2022; Joaquim, Juzwiak & Winckler, 2019; Eskici & Ersoy, 2016). Particularly regarding plant-based and carbohydrate-rich foods. Fruit and vegetable consumption was especially low, with only 7% and 3% of players meeting the respective recommendations, while more than 85% consumed fewer cereal servings than advised. This low intake of carbohydrate-rich foods may compromise athletic performance, as previously suggested in athletes with spinal cord injury (Figel et al., 2018). In WCB players, Ferro et al. (2017) reported carbohydrate intake at approximately 45% of total energy, while Toti et al. (2022) described inadequate energy intake and macronutrient distribution. More recently, Urhan et al. (2025) reported poor diet quality and low energy availability among Paralympic basketball athletes, largely related to insufficient carbohydrate intake.

Similarly, Sasaki & da Costa (2023) and Ghazzawi et al. (2025) identified low intake of plant-based foods, insufficient carbohydrate intake, excessive dietary fat, micronutrient deficiencies, and suboptimal hydration as common issues among para-athletes. These findings reinforce the need for food-based nutritional strategies aimed at increasing the intake of fruits, vegetables, legumes, cereals, and other carbohydrate-rich foods adapted to the training demands and functional characteristics of WCB players.

Overall, previous literature and the present results converge in showing insufficient intake of foods rich in complex carbohydrates and fiber, such as fruits, vegetables, legumes, and cereals (Broad, 2019; Thomas, Erdman & Burke, 2016). This dietary pattern not only limits essential micronutrient intake but may also reduce total energy availability, particularly in the context of high training loads or intense competition (Grabia et al., 2024). Low carbohydrate intake has been associated with an increased risk of relative energy deficiency in sport (RED-S), which negatively affects health and performance, through hormonal, menstrual, bone, immune, gastrointestinal, and psychological disturbances, while also increasing the risk of injury and disordered eating behaviors (Mountjoy et al., 2018). It is important to note that athletes with physical impairments, particularly those with spinal cord injury, may exhibit lower absolute energy expenditure than able-bodied athletes due to reduced active muscle mass and altered autonomic responses. Nevertheless, despite lower total energy requirements, appropriate macronutrient distribution, especially carbohydrate availability, remains crucial to maintain training quality and performance (Mountjoy et al., 2018; Kerksick et al., 2018).

Similarly, the low consumption of fruits, vegetables, legumes, and cereals entails insufficient fiber intake, which in individuals with reduced mobility may increase the risk of constipation (Toti et al., 2022). Adequate fiber intake has been widely recommended in wheelchair users—both athletes and non-athletes, to support proper bowel function (Goosey-Tolfrey & Crosland, 2010). Importantly, a substantial proportion of Paralympic athletes with spinal cord injury (SCI) may present gastrointestinal complications such as neurogenic bowel dysfunction (NBD), which can be more prevalent and clinically relevant in this group than athletes with other impairments such as amputations. Comparative evidence in Paralympic populations indicates that athletes with SCI show a higher burden of bowel-related dysfunctions, reflecting the impact of impaired neural control of gastrointestinal function (Peluso et al., 2025). NBD is characterized by reduced colonic motility and prolonged intestinal transit time, which in some cases may extend up to approximately 80 h, leading to an increased risk of constipation and related complications (Ghazzawi et al., 2025). These alterations have important nutritional implications, as athletes with SCI may require specific dietary strategies to support gastrointestinal function and overall health. In particular, current evidence highlights the need for tailored nutritional interventions, including adequate intake of dietary fiber and fluids, as well as individualized dietary planning to address altered metabolism, impaired gastrointestinal function, and hydration challenges in this population (Ghazzawi et al., 2025).

Protein-related food consumption also showed an imbalanced profile, with low intake of legumes and fish and excessive red meat consumption. Although previous studies suggest that para-athletes may meet total protein recommendations (Figel et al., 2018; Madden, Shearer & Parnell, 2017), protein sources should also be considered. In the present study, 78% of participants consumed more red meat than recommended. This pattern is consistent with Toti et al. (2022), who reported that approximately 66% of protein intake among WCB players derived from animal sources. Replacing part of the red and processed meat intake with legumes, nuts, fish, eggs, dairy products, and other high-quality protein sources could improve dietary balance and cardiometabolic profile (Flueck & Parnell, 2021; Ghazzawi et al., 2025).

Regarding water intake, most players failed to meet the minimum recommended requirements for the general population. Low hydration levels can directly impair athletic performance (Thomas, Erdman & Burke, 2016) and may be particularly relevant in athletes with physical impairments because thermoregulation can be affected by impairment type. In amputee athletes, reduced body surface area and heat accumulation between the prosthesis and residual limb may compromise heat dissipation (Andrews et al., 2016). In athletes with spinal cord injury, impaired thermoregulation, lower sweating rates, and reduced heat dissipation capacity may hinder effective core temperature control, and increase fatigue of risk (Pritchett et al., 2020). These findings support the need to include individualized hydration strategies in nutritional planning for WCB players. Therefore, the low consumption of fiber-rich foods and suboptimal hydration patterns observed in the present study may have clinically relevant consequences, especially among athletes with SCI, reinforcing the need for targeted, impairment-specific nutritional approaches (Ghazzawi et al., 2025).

These patterns are supported by the analysis of the DDS, which provides a synthetic indicator of overall dietary variety. Dietary diversity has been widely used as a proxy indicator of dietary quality and nutrient adequacy, as higher DDS values are generally associated with more balanced diets and improved micronutrient intake. In our study, the DDS score showed very low variability among participants, which indirectly suggests a lack of dietary variety (Kojima, Murayama & Suga, 2020; Ruel, 2003). Consequently, this finding may point to insufficient micronutrient intake, as monotonous diets (characterized by low diversity) are less likely to meet the recommended daily intakes of essential vitamins and minerals.

### Strengths of the study

One of the main strengths of this study is its focus on a specific and under-researched population: Spanish WCB players competing at a high level. The inclusion of athletes from a high proportion of Spanish first-division WCB teams increases the relevance of the findings within this sporting context.

Another strength is the food-group-based approach. While previous research in Paralympic athletes has mainly focused on energy and nutrient intake (Toti et al., 2022; Joaquim, Juzwiak & Winckler, 2019; Eskici & Ersoy, 2016), this perspective provides practical information for identifying dietary behaviors that can be addressed through nutritional education and individualized interventions. Furthermore, the use of a previously validated Food4Me FFQ also increases the methodological consistency of the dietary assessment.

### Practical applications and recommendations

The findings have practical implications for athletes, coaches, nutritionists, and multidisciplinary teams. Nutritional strategies should prioritize increasing the intake of fruit, vegetables, legumes, whole-grain cereals, fish, nuts, and water, while reducing excessive consumption of red and processed meat (Toti et al., 2022; Urhan et al., 2025; Kerksick et al., 2018). Nutrition should be integrated into the regular training environment through individualized assessments should consider sex, functional classification, type of impairment, gastrointestinal function, body composition goals, training load, competition schedule, and personal preferences, with special attention to carbohydrate availability, hydration, fiber intake, and the quality of protein and fat sources.

### Limitations

This study has some limitations. The main limitation is the restricted sample size, particularly the low representation of female athletes (*n* = 9), which limits the interpretation of sex-based comparisons and precludes meaningful analyses according to functional category in women. Although athletes from 83% of Spanish first-division WCB teams were assessed and the statistical power calculation was satisfactory, the use of a non-probabilistic convenience sampling method represents a potential source of selection bias, as voluntary participants might have a higher interest in nutrition. Thus, caution is needed when extrapolating these results to other athletic groups.

Regarding the methodology, the use of FFQ allowed a rapid, cost-effective, and minimally disruptive estimation of typical dietary intake. However, the data were self-reported and retrospective, and therefore subject to recall bias, social desirability bias, and potential inaccuracies. Moreover, while the FFQ was previously validated and useful for characterizing eating patterns, it was not specifically designed for athletic populations and does not allow precise quantification of portion sizes or nutrients. Notably, it may exclude sport-specific items such as energy gels or bars; however, this information was collected through a separate instrument and is being analyzed in a parallel investigation to provide a dedicated evaluation of sports supplements.

Finally, the cross-sectional design of this study precludes the establishment of causal interpretations, providing only a snapshot of the current situation. Since dietary habits may change over time due to training load, nutritional advice, or body composition goals, periodic assessments would be necessary to capture changes in habitual intake.

### Future lines of research

Future research should include larger samples of wheelchair athletes, with greater representation of female players and athletes from different sports and impairment types. Longitudinal studies are also needed to evaluate changes in dietary habits across training and competition periods. In addition, future studies should estimate total energy, macronutrient, and micronutrient intake from food consumption and assess the effectiveness of tailored nutritional interventions aimed at improving diet quality, hydration practices, gastrointestinal health, and sports performance in athletes with physical impairments.

## Conclusions

The results of the present study showed low compliance with dietary recommendations across most food groups, particularly fruits, vegetables, legumes, cereals, and fish, whereas adherence was adequate only for dairy products and eggs. Conversely, red meat consumption exceeded recommended levels. This pattern reflects an overall suboptimal diet quality, which may compromise energy availability and, consequently, athletic performance.

Although differences were hypothesized in food-consumption habits by sex and functional classification, significant differences were found only in dairy intake, which was lower among women and athletes with greater functional limitation. The findings also confirmed that most players did not follow any specific diet, and among those who did, the main motivations were performance improvement and health maintenance. Furthermore, the majority of participants failed to meet the recommended food-frequency guidelines.

Overall, these results indicate that WCB players show limited adherence to healthy-eating patterns, consistent with findings in other athletes with physical disabilities. The study underscores the need to implement nutrition education and counseling programs tailored to sex, functional classification, and competitive context, with the goal of promoting a more balanced dietary pattern that meets the energetic and physiological demands of this population.

## Supplemental Information

10.7717/peerj.21715/supp-1 Supplemental Information 1 Dietary Intake, Guideline Adherence, and Hydration in Elite Wheelchair Basketball Players. Supplementary tables reporting food group consumption frequencies, dietary recommendation adherence, and water intake patterns in elite wheelchair basketball players, stratified by sex and functional level, with associated chi-square statistics and effect sizes

10.7717/peerj.21715/supp-2 Supplemental Information 2 STROBE Checklist.

## Additional Information and Declarations

### Data Availability

The following information was supplied regarding data availability:

The raw data is available at Zenodo:

- Norte, A., Miana, M., Romero García, D., Sospedra, I., Ausó, E., Cifuentes, A., Perpiñá Martínez, S., Aprea Moreno, S., Leardy Palencia, P., Delgado Ortiz, S., Martínez Sanz, J. M.& BUCETA TORO, M. I. (2026). Food intake in Spanish elite wheelchair basketball players [Data set]. Zenodo. https://doi.org/10.5281/zenodo.20834982.
