# Supplementary material for: Eating for performance: an evaluation of food consumption patterns and adherence to dietary guidelines in elite wheelchair basketball players
Source: PeerJ. 2026 Sep 16;14:e21715. doi: 10.7717/peerj.21715 (PMC13589512; doi:10.7717/peerj.21715)
Supplement: Supplementary file 1 [file peerj-14-21715-s001.docx]

Supplementary tables

Supplementary Table S1 shows the frequencies of consumption of different food groups according to sex. The only significant difference found was in the consumption of soft drinks (p = 0.031), where women showed proportionally higher consumption compared with men. No significant differences were found for the remaining food groups (p = 0.052–0.669).

Table S1. Frequency (%) of food group consumption according to sex

| Ítems | **Sex** | | | | | | | | | | | | | | |
| --- | --- | --- | --- | --- | --- | --- | --- | --- | --- | --- | --- | --- | --- | --- | --- |
|  | **Female (n = 9)** | | | | | | **Male (n = 57)** | | | | | | **Statistical values** | | |
|  | **<1 t/w** | **1-2 t/w** | **3-4 t/w** | **5-6 t/w** | **1-2 t/d** | **>3 t/d** | **<1 t/w** | **1-2 t/w** | **3-4 t/w** | **5-6 t/w** | **1-2 t/d** | **>3 t/d** | **X^2^** | ***p*** | **V** |
| Fruit | 1 (11.1%) | 3 (33.3%) | 1 (11.1%) | 2 (22.2%) | 2 (22.2%) | 0 (0.0%) | 4 (7.0%) | 5 (8.8%) | 19 (33.3%) | 7 (12.3%) | 17 (29.8%) | 5 (8.8%) | 6.816 | 0.235 | 0.321 |
| *Residual* | *0.4* | *2.1* | *-1.3* | *0.8* | *-0.5* | *-0.9* | *-0.4* | *-2.1* | *1.3* | *-0.8* | *0.5* | *0.9* |  |  |  |
| Vegetables | 0 (0.0%) | 3 (33.3%) | 0 (0.0%) | 2 (22.2%) | 4 (44.4%) | 0 (0.0%) | 2 (3.5%) | 9 (15.8%) | 19 (33.3%) | 11 (19.3%) | 14 (24.6%) | 2 (3.5%) | 6.108 | 0.296 | 0.304 |
| *Residual* | *-0.6* | *1.3* | *-2.1* | *0.2* | *1.2* | *-0.6* | *0.6* | *-1.3* | *2.1* | *-0.2* | *-1.2* | *0.6* |  |  |  |
| Specific vegetables | 3 (33.3%) | 2 (22.2%) | 2 (22.2%) | 0 (0.0%) | 2 (22.2%) | 0 (0.0%) | 20 (35.1%) | 19 (33.3%) | 9 (15.8%) | 6 (10.5%) | 3 (5.3%) | 0 (0.0%) | 4.400 | 0.355 | 0.258 |
| *Residual* | *-0.1* | *-0.7* | *0.5* | *-1.0* | *1.8* | *0.0* | *0.1* | *0.7* | *-0.5* | *1.0* | *-1.8* | *0.0* |  |  |  |
| Legumes | 1 (11.1%) | 7 (77.8%) | 0 (0.0%) | 1 (11.1%) | 0 (0.0%) | 0 (0.0%) | 13 (22.8%) | 25 (43.9%) | 14 (24.6%) | 3 (5.3%) | 2 (3.5%) | 0 (0.0%) | 5.310 | 0.257 | 0.284 |
| *Residual* | *-0.8* | *1.9* | *-1.7* | *0.7* | *-0.6* | *0.0* | *0.8* | *-1.9* | *1.7* | *-0.7* | *0.6* | *0.0* |  |  |  |
| Tubers | 1 (11.1%) | 3 (33.3%) | 3 (33.3%) | 1 (11.1%) | 1 (11.1%) | 0 (0.0%) | 5 (8.8%) | 23 (40.4%) | 23 (40.4%) | 6 (10.5%) | 0 (0.0%) | 0 (0.0%) | 6.577 | 0.160 | 0.316 |
| *Residual* | *0.2* | *-0.4* | *-0.4* | *0.1* | *2.5* | *0.0* | *-0.2* | *0.4* | *0.4* | *-0.1* | *-2.5* | *0.0* |  |  |  |
| Pasta | 0 (0.0%) | 4 (44.4%) | 3 (33.3%) | 2 (22.2%) | 0 (0.0%) | 0 (0.0%) | 6 (10.5%) | 16 (28.1%) | 25 (43.9%) | 7 (12.3%) | 3 (5.3%) | 0 (0.0%) | 2.875 | 0.579 | 0.209 |
| *Residual* | *-1.0* | *1.0* | *-0.6* | *0.8* | *-0.7* | *0.0* | *1.0* | *-1.0* | *0.6* | *-0.8* | *0.7* | *0.0* |  |  |  |
| Rice | 3 (33.3%) | 4 (44.4%) | 2 (22.2%) | 0 (0.0%) | 0 (0.0%) | 0 (0.0%) | 4 (7.0%) | 28 (49.1%) | 13 (22.8%) | 8 (14.0%) | 4 (7.0%) | 0 (0.0%) | 7.006 | 0.136 | 0.326 |
| *Residual* | *2.4* | *-0.3* | *0.0* | *-1.2* | *-0.8* | *0.0* | *-2.4* | *0.3* | *0.0* | *1.2* | *0.8* | *0.0* |  |  |  |
| Bread | 3 (33.3%) | 3 (33.3%) | 1 (11.1%) | 2 (22.2%) | 0 (0.0%) | 0 (0.0%) | 10 (17.5%) | 13 (22.8%) | 16 (28.1%) | 8 (14.0%) | 9 (15.8%) | 1 (1.8%) | 4.130 | 0.531 | 0.250 |
| *Residual* | *1.1* | *0.7* | *-1.1* | *0.6* | *-1.3* | *-0.4* | *-1.1* | *-0.7* | *1.1* | *-0.6* | *1.3* | *0.4* |  |  |  |
| Whole grains | 4 (44.4%) | 2 (22.2%) | 1 (11.1%) | 1 (11.1%) | 1 (11.1%) | 0 (0.0%) | 16 (28.1%) | 20 (35.1%) | 10 (17.5%) | 9 (15.8%) | 2 (3.5%) | 0 (0.0%) | 2.367 | 0.669 | 0.189 |
| Residual | 1.0 | -0.8 | -0.5 | -0.4 | 1.0 | *0.0* | -1.0 | 0.8 | 0.5 | 0.4 | -1.0 | *0.0* |  |  |  |
| White meat | 0 (0.0%) | 2 (22.2%) | 6 (66.7%) | 1 (11.1%) | 0 (0.0%) | 0 (0.0%) | 2 (3.5%) | 11 (19.3%) | 23 (40.4%) | 17 (29.8%) | 4 (7.0%) | 0 (0.0%) | 3.204 | 0.524 | 0.220 |
| *Residual* | *-0.6* | *0.2* | *1.5* | *-1.2* | *-0.8* | *0.0* | *0.6* | *-0.2* | *-1.5* | *1.2* | *0.8* | *0.0* |  |  |  |
| Eggs | 0 (0.0%) | 5 (55.6%) | 2 (22.2%) | 1 (11.1%) | 1 (11.1%) | 0 (0.0%) | 5 (8.8%) | 8 (14.0%) | 25 (43.9%) | 15 (26.3%) | 4 (7.0%) | 0 (0.0%) | 9.395 | 0.052 | 0.377 |
| *Residual* | *-0.9* | *2.9* | *-1.2* | *-1.0* | *0.4* | *0.0* | *0.9* | *-2.9* | *1.2* | *1.0* | *-0.4* | *0.0* |  |  |  |
| Fish | 2 (22.2%) | 7 (77.8%) | 0 (0.0%) | 0 (0.0%) | 0 (0.0%) | 0 (0.0%) | 15 (26.3%) | 32 (56.1%) | 8 (14.0%) | 2 (3.5%) | 0 (0.0%) | 0 (0.0%) | 2.245 | 0.523 | 0.184 |
| *Residual* | *-0.3* | *1.2* | *-1.2* | *-0.6* | *0.0* | *0.0* | *0.3* | *-1.2* | *1.2* | *0.6* | *0.0* | *0.0* |  |  |  |
| Red meat | 3 (33.3%) | 4 (44.4%) | 2 (22.2%) | 0 (0.0%) | 0 (0.0%) | 0 (0.0%) | 9 (15.8%) | 20 (35.1%) | 26 (45.6%) | 2 (3.5%) | 0 (0.0%) | 0 (0.0%) | 2.821 | 0.420 | 0.207 |
| *Residual* | *1.3* | *0.5* | *-1.3* | *-0.6* | *0.0* | *0.0* | *-1.3* | *-0.5* | *1.3* | *0.6* | *0.0* | *0.0* |  |  |  |
| Nuts | 5 (55.6%) | 1 (11.1%) | 0 (0.0%) | 2 (22.2%) | 1 (11.1%) | 0 (0.0%) | 16 (28.1%) | 20 (35.1%) | 12 (21.1%) | 5 (8.8%) | 3 (5.3%) | 1 (1.8%) | 7.067 | 0.216 | 0.327 |
| *Residual* | *1.6* | *-1.4* | *-1.5* | *1.2* | *0.7* | *-0.4* | *-1.6* | *1.4* | *1.5* | *-1.2* | *-0.7* | *0.4* |  |  |  |
| Fermented foods | 4 (44.4%) | 2 (22.2%) | 3 (33.3%) | 0 (0.0%) | 0 (0.0%) | 0 (0.0%) | 22 (38.6%) | 11 (19.3%) | 12 (21.1%) | 10 (17.5%) | 2 (3.5%) | 0 (0.0%) | 2.512 | 0.643 | 0.195 |
| *Residual* | *0.3* | *0.2* | *0.8* | *-1.4* | *-0.6* | *0.0* | *-0.3* | *-0.2* | *-0.8* | *1.4* | *0.6* | *0.0* |  |  |  |
| Soft drinks | 3 (33.3%) | 1 (11.1%) | 0 (0.0%) | 3 (33.3%) | 2 (22.2%) | 0 (0.0%) | 24 (42.1%) | 17 (29.8%) | 8 (14.0%) | 4 (7.0%) | 2 (3.5%) | 2 (3.5%) | 12.290 | 0.031 | 0.432 |
| *Residual* | *-0.5* | *-1.2* | *-1.2* | *2.4* | *2.2* | *-0.6* | *0.5* | *1.2* | *1.2* | *-2.4* | *-2.2* | *0.6* |  |  |  |
| Commercial sweets | 3 (33.3%) | 1 (11.1%) | 3 (33.3%) | 1 (11.1%) | 1 (11.1%) | 0 (0.0%) | 17 (29.8%) | 21 (36.8%) | 15 (26.3%) | 3 (5.3%) | 0 (0.0%) | 1 (1.8%) | 8.646 | 0.124 | 0.362 |
| *Residual* | *0.2* | *-1.5* | *0.4* | *0.7* | *2.5* | *-0.4* | *-0.2* | *1.5* | *-0.4* | *-0.7* | *-2.5* | *0.4* |  |  |  |
| Prep/froz foods | 4 (44.4%) | 5 (55.6%) | 0 (0.0%) | 0 (0.0%) | 0 (0.0%) | 0 (0.0%) | 30 (52.6%) | 19 (33.3%) | 5 (8.8%) | 3 (5.3%) | 0 (0.0%) | 0 (0.0%) | 2.420 | 0.490 | 0.191 |
| *Residual* | *-0.5* | *1.3* | *-0.9* | *-0.7* | *0.0* | *0.0* | *0.5* | *-1.3* | *0.9* | *0.7* | *0.0* | *0.0* |  |  |  |
| Dairy products | 2 (22.2%) | 1 (11.1%) | 1 (11.1%) | 1 (11.1%) | 4 (44.4%) | 0 (0.0%) | 11 (19.3%) | 16 (28.1%) | 9 (15.8%) | 13 (22.8%) | 7 (12.3%) | 1 (1.8%) | 6.498 | 0.261 | 0.314 |
| *Residual* | *0.2* | *-1.1* | *-0.4* | *-0.8* | *2.4* | *-0.4* | *-0.2* | *1.1* | *0.4* | *0.8* | *-2.4* | *0.4* |  |  |  |
| Plant based beverages | 5 (55.6%) | 2 (22.2%) | 0 (0.0%) | 1 (11.1%) | 1 (11.1%) | 0 (0.0%) | 41 (71.9% | 11 (19.3%) | 1 (1.8%) | 1 (1.8%) | 2 (3.5%) | 1 (1.8%) | 3.882 | 0.566 | 0.243 |
| *Residual* | *-1.0* | *0.2* | *-0.4* | *1.5* | *1.0* | *-0.4* | *1.0* | *-0.2* | *0.4* | *-1.5* | *-1.0* | *0.4* |  |  |  |

t/w: time/week; t/d: time/day; χ²: chi-square test value; V: Cramer’s V; prep/froz: prepared/frozen foods.

Supplementary Table S2 presents the frequencies of consumption of the different food groups according to functional classification. The only significant difference observed was in dairy consumption (p = 0.022), with athletes classified as functionality grade 4 showing proportionally lower consumption, while those classified as grade 1 showed proportionally higher consumption. No significant differences were found for the remaining food groups (p = 0.133–0.894).

Table S2. Frequency (%) of food group consumption according to functional classification.

| Ítems | | | Fruit | Vegetables | Specific vegetables | Legumes | Tubers | Pasta | Rice | Bread | Whole grains | White meat | Eggs | Fish | Red meat | Nuts | Fermented foods | Soft drinks | Comercial Sweeets | Prep/foz foods | Dairy products | Plant-based beverages |
| --- | --- | --- | --- | --- | --- | --- | --- | --- | --- | --- | --- | --- | --- | --- | --- | --- | --- | --- | --- | --- | --- | --- |
| **Finctional level** | **Gr 1 (n = 14)** | **<1 t/w** | 2 (14.3%) | 1 (7.1%) | 7 (50.0%) | 3 (21.4%) | 1 (7.1%) | 3 (21.4%) | 4 (28.6%) | 2 (14.3%) | 7 (50.0%) | 0 (0.0%) | 0 (0.0%) | 4 (28.6%) | 1 (7.1%) | 5 (35.7%) | 5 (35.7%) | 6 (42.9%) | 3 (21.4%) | 7 (50.0%) | 3 (21.4%) | 12 (85.7%) |
|  |  | Rs | 1.1 | 1.0 | 1.3 | 0.0 | -0.3 | 1.8 | 2.5 | -0.6 | 1.8 | -0.7 | -1.2 | 0.3 | -1.2 | 0.4 | -0.3 | 0.2 | -0.8 | -0.1 | 0.2 | 1.5 |
|  |  | **1-2 t/w** | 1 (7.1%) | 5 (35.7%) | 4 (28.6%) | 8 (57.1%) | 6 (42.9%) | 4 (28.6%) | 7 (50.0%) | 4 (28.6%) | 6 (42.9%) | 6 (42.9%) | 4 (28.6%) | 9 (64.3%) | 7 (50.0%) | 5 (35.7%) | 4 (28.6%) | 2 (14.3%) | 6 (42.9%) | 5 (35.7%) | 3 (21.4%) | 1 (7.1%) |
|  |  | Rs | -0.6 | 1.9 | -0.3 | 0.7 | 0.3 | -0.2 | 0.1 | 0.4 | 0.9 | 2.5 | 0.9 | 0.4 | 1.2 | 0.4 | 0.9 | -1.2 | 0.9 | -0.1 | -0.4 | -1.3 |
|  |  | **3-4 t/w** | 4 (28.6%) | 3 (21.4%) | 1 (7.1%) | 3 (21.4%) | 6 (42.9%) | 6 (42.9%) | 3 (21.4%) | 5 (35.7%) | 0 (0.0%) | 5 (35.7%) | 5 (35.7%) | 1 (7.1%) | 5 (35.7%) | 3 (21.4%) | 3 (21.4%) | 2 (14.3%) | 3 (21.4%) | 2 (14.3%) | 2 (14.3%) | 0 (0.0%) |
|  |  | Rs | -0.2 | -0.7 | -1.1 | 0.0 | 0.3 | 0 | -0.1 | 1.0 | -1.9 | -0.7 | -0.4 | -0.6 | -0.6 | 0.4 | -0.1 | 0.3 | -0.6 | 1.1 | -0.1 | -0.5 |
|  |  | **5-6 t/w** | 0 (0.0%) | 1 (7.1%) | 0 (0.0%) | 0 (0.0%) | 0 (0.0%) | 0 (0.0%) | 0 (0.0%) | 2 (14.3%) | 1 (7.1%) | 2 (14.3%) | 4 (28.6%) | 0 (0.0%) | 1 (7.1%) | 1 (7.1%) | 2 (14.3%) | 2 (14.3%) | 1 (7.1%) | 0 (0.0%) | 0 (0.0%) | 0 (0.0%) |
|  |  | R | -1.7 | -1.3 | -1.3 | -1.1 | -1.5 | -1.7 | -1.6 | -0.1 | -0.9 | -1.2 | 0.4 | -0.7 | 1.0 | -0.5 | -0.1 | 0.5 | 0.2 | -0.9 | -2.2 | -0.7 |
|  |  | **1-2 t/d** | 7 (50.0%) | 4 (28.6%) | 2 (14.3%) | 0 (0.0%) | 1 (7.1%) | 1 (7.1%) | 0 (0.0%) | 1 (7.1%) | 0 (0.0%) | 1 (7.1%) | 1 (7.1%) | 0 (0.0%) | 0 (0.0%) | 0 (0.0%) | 0 (0.0%) | 2 (14.3%) | 1 (7.1%) | 0 (0.0%) | 5 (35.7%) | 1 (7.1%) |
|  |  | Rs | 2.0 | 0.1 | 1.1 | -0.7 | 1.9 | 0.5 | -1.1 | -0.8 | -0.9 | 0.2 | -0.1 | 0.0 | 0.0 | -1.1 | -0.7 | 1.5 | 1.9 | 0.0 | 2.2 | 0.5 |
|  |  | **>3 t/d** | 0 (0.0%) | 0 (0.0%) | 0 (0.0%) | 0 (0.0%) | 0 (0.0%) | 0 (0.0%) | 0 (0.0%) | 0 (0.0%) | 0 (0.0%) | 0 (0.0%) | 0 (0.0%) | 0 (0.0%) | 0 (0.0%) | 0 (0.0%) | 0 (0.0%) | 0 (0.0%) | 0 (0.0%) | 0 (0.0%) | 1 (7.1%) | 0 (0.0%) |
|  |  | Rs | -1.2 | -0.7 | 0.0 | 0.0 | 0.0 | 0.0 | 0.0 | -0.5 | 0.0 | 0.0 | 0.0 | 0.0 | 0.0 | -0.5 | 0.0 | -0.7 | -0.5 | 0.0 | 1.9 | -0.5 |
|  | **Gr 2 (n = 10)** | **<1 t/w** | 0 (0.0%) | 0 (0.0%) | 3 (30.0%) | 4 (40.0%) | 0 (0.0%) | 0 (0.0%) | 1 (10.0%) | 3 (30.0%) | 4 (40.0%) | 0 (0.0%) | 1 (10.0%) | 3 (30.0%) | 4 (40.0%) | 6 (60.0%) | 5 (50.0%) | 4 (40.0%) | 4 (40.0%) | 6 (60.0%) | 1 (7.1%) | 7 (70.0%) |
|  |  | Rs | -1.0 | -0.6 | -0.3 | 1.6 | -1.1 | -1.1 | -0.1 | 0.9 | 0.7 | -0.6 | 0.3 | 0.3 | 1.9 | 2.1 | 0.7 | -0.1 | 0.7 | 0.6 | -0.8 | 0.0 |
|  |  | **1-2 t/w** | 3 (30.0%) | 2 (20.0%) | 4 (40.0%) | 3 (30.0%) | 3 (30.0%) | 2 (20.0%) | 4 (40.0%) | 3 (30.0%) | 4 (40.0%) | 3 (30.0%) | 2 (20.0%) | 6 (60.0%) | 3 (30.0%) | 2 (20.0%) | 0 (0.0%) | 3 (30.0%) | 3 (30.0%) | 3 (30.0%) | 1 (7.1%) | 3 (30.0%) |
|  |  | Rs | 1.9 | 0.3 | 0.6 | -1.3 | -0.7 | -0.8 | -0.6 | 0.5 | 0.5 | -0.8 | 0.0 | 0.1 | -0.5 | -0.9 | -1.7 | 0.2 | -0.2 | -0.5 | -1.2 | 0.9 |
|  |  | **3-4 t/w** | 3 (30.0%) | 2 (20.0%) | 1 (10.0%) | 2 (20.0%) | 4 (40.0%) | 5 (50.0%) | 4 (40.0%) | 1 (10.0%) | 0 (0.0%) | 1 (10.0%) | 4 (40.0%) | 1 (10.0%) | 3 (30.0%) | 2 (20.0%) | 1 (10.0%) | 1 (10.0%) | 3 (30.0%) | 1 (10.0%) | 3 (30.0%) | 0 (0.0%) |
|  |  | Rs | 0.0 | -0.7 | -0.6 | -0.1 | 0.0 | 0.5 | 1.4 | -1.2 | -1.5 | -1.0 | -0.1 | -0.2 | -0.9 | 0.2 | -1.0 | -0.2 | 0.2 | 0.3 | 1.4 | -0.4 |
|  |  | **5-6 t/w** | 2 (20.0%) | 2 (20.0%) | 2 (20.0%) | 1 (10.0%) | 3 (30.0%) | 2 (20.0%) | 1 (10.0%) | 2 (20.0%) | 2 (20.0%) | 5 (50.0%) | 2 (20.0%) | 0 (0.0%) | 0 (0.0%) | 0 (0.0%) | 4 (19.0%) | 1 (10.0%) | 0 (0.0%) | 0 (0.0%) | 4 (40.0%) | 0 (0.0%) |
|  |  | Rs | 0.6 | 0.0 | 1.3 | 0.6 | 2.2 | 0.6 | -0.2 | 0.5 | 0.5 | 1.8 | -0.3 | -0.6 | -0.6 | -1.2 | 2.4 | -0.1 | -0.9 | -0.7 | 1.6 | -0.6 |
|  |  | **1-2 t/d** | 1 (10.0%) | 3 (30.0%) | 0 (0.0%) | 0 (0.0%) | 0 (0.0%) | 1 (10.0%) | 0 (0.0%) | 0 (0.0%) | 0 (0.0%) | 1 (10.0%) | 1 (10.0%) | 0 (0.0%) | 0 (0.0%) | 0 (0.0%) | 0 (0.0%) | 0 (0.0%) | 0 (0.0%) | 0 (0.0%) | 1 (7.1%) | 0 (0.0%) |
|  |  | Rs | -1.4 | 0.2 | -1.0 | -0.6 | -0.4 | 0.9 | -0.9 | -1.4 | -0.7 | 0.6 | 0.3 | 0.0 | 0.0 | -0.9 | -0.6 | -0.9 | -0.4 | 0.0 | -0.6 | -0.7 |
|  |  | **>3 t/d** | 1 (10.0%) | 1 (10.0%) | 0 (0.0%) | 0 (0.0%) | 0 (0.0%) | 0 (0.0%) | 0 (0.0%) | 1 (10.0%) | 0 (0.0%) | 0 (0.0%) | 0 (0.0%) | 0 (0.0%) | 0 (0.0%) | 0 (0.0%) | 0 (0.0%) | 1 (10.0%) | 0 (0.0%) | 0 (0.0%) | 0 (0.0%) | 0 (0.0%) |
|  |  | Rs | 0.3 | 1.4 | 0.0 | 0.0 | 0.0 | 0.0 | 0.0 | 2.4 | 0.0 | 0.0 | 0.0 | 0.0 | 0.0 | -0.4 | 0.0 | 1.4 | -0.4 | 0.0 | -0.4 | -0.4 |
|  | **Gr 3 (n = 21)** | **<1 t/w** | 2 (9.5%) | 0 (0.0%) | 7 (33.3%) | 3 (14.3%) | 2 (9.5%) | 1 (4.8%) | 0 (0.0%) | 4 (19.0%) | 3 (14.3%) | 2 (9.5%) | 2 (9.5%) | 5 (23.8%) | 4 (19.0%) | 5 (23.8%) | 7 (33.3%) | 9 (42.9%) | 7 (33.3%) | 9 (42.9%) | 1 (4.8%) | 13 (61.9%) |
|  |  | Rs | 0.4 | -1.0 | -0.2 | -0.9 | 0.1 | -0.8 | -1.9 | -0.1 | -1.9 | 2.1 | 0.4 | -0.2 | 0.1 | -1.0 | -0.7 | 0.2 | 0.4 | -1.0 | -2.1 | 0.0 |
|  |  | **1-2 t/w** | 1 (4.8%) | 3 (14.3%) | 6 (28.6%) | 11 (52.4%) | 7 (33.3%) | 8 (38.1%) | 13 (61.9%) | 4 (19.0%) | 7 (33.3%) | 1 (4.8%) | 5 (23.8%) | 14 (66.7%) | 10 (47.6%) | 7 (33.3%) | 4 (19.0%) | 7 (33.3%) | 3 (14.3%) | 8 (38.1%) | 7 (33.3%) | 5 (23.8%) |
|  |  | Rs | -1.3 | -0.6 | -0.4 | 0.4 | -0.7 | 0.9 | 1.5 | -0.7 | 0.0 | -2.1 | 0.6 | 0.9 | 1.3 | 0.2 | -0.1 | 0.8 | -2.2 | 0.2 | 1.0 | 0.9 |
|  |  | **3-4 t/w** | 7 (33.3%) | 9 (42.9%) | 6 (28.6%) | 6 (28.6%) | 10 (47.6%) | 9 (42.9%) | 4 (19.0%) | 5 (23.8%) | 6 (28.6%) | 12 (57.1%) | 6 (28.6%) | 2 (9.5%) | 7 (33.3%) | 6 (28.6%) | 6 (28.6%) | 3 (14.3%) | 10 (47.6%) | 2 (9.5%) | 5 (23.8%) | 1 (4.8%) |
|  |  | Rs | 0.4 | 1.7 | 1.8 | 1.0 | 0.9 | 0.0 | -0.5 | -0.2 | 1.8 | 1.5 | -1.4 | -0.4 | -1.0 | 1.5 | 0.8 | 0.4 | 2.5 | 0.4 | 1.3 | -0.4 |
|  |  | **5-6 t/w** | 4 (19.0%) | 4 (19.0%) | 1 (4.8%) | 0 (0.0%) | 2 (9.5%) | 3 (14.3%) | 2 (9.5%) | 4 (19.0%) | 3 (14.3%) | 6 (28.6%) | 6 (28.6%) | 0 (0.0%) | 0 (0.0%) | 1 (4.8%) | 2 (9.5%) | 1 (4.8%) | 0 (0.0%) | 2 (9.5%) | 4 (19.0%) | 0 (0.0%) |
|  |  | Rs | 0.9 | -0.1 | -0.8 | -1.4 | -0.2 | 0.1 | -0.4 | 0.6 | -0.1 | 0.2 | 0.6 | -1.0 | -1.0 | -1.1 | -0.9 | -1.1 | -1.4 | 1.3 | -0.3 | -0.6 |
|  |  | **1-2 t/d** | 5 (23.8%) | 5 (23.8%) | 1 (4.8%) | 1 (4.8%) | 0 (0.0%) | 0 (0.0%) | 2 (9.5%) | 4 (19.0%) | 2 (9.5%) | 0 (0.0%) | 2 (9.5%) | 0 (0.0%) | 0 (0.0%) | 1 (4.8%) | 2 (9.5%) | 0 (0.0%) | 0 (0.0%) | 0 (0.0%) | 4 (19.0%) | 1 (4.8%) |
|  |  | Rs | -0.6 | -0.4 | -0.6 | 0.6 | -0.7 | -1.2 | 0.8 | 0.9 | 1.3 | -1.4 | 0.4 | 0.0 | 0.0 | -0.3 | 2.1 | -1.4 | -0.7 | 0.0 | 0.4 | -0.7 |
|  |  | **>3 t/d** | 2 (9.5%) | 0 (0.0%) | 0 (0.0%) | 0 (0.0%) | 0 (0.0%) | 0 (0.0%) | 0 (0.0%) | 0 (0.0%) | 0 (0.0%) | 0 (0.0%) | 0 (0.0%) | 0 (0.0%) | 0 (0.0%) | 1 (4.8%) | 0 (0.0%) | 1 (4.8%) | 1 (4.8%) | 0 (0.0%) | 0 (0.0%) | 1 (4.8%) |
|  |  | Rs | 0.4 | -1.0 | 0.0 | 0.0 | 0.0 | 0.0 | 0.0 | -0.7 | 0.0 | 0.0 | 0.0 | 0.0 | 0.0 | 1.5 | 0.0 | 0.6 | 1.5 | 0.0 | -0.7 | -0.4 |
|  | **Gr 4 (n = 21)** | **<1 t/w** | 1 (4.8%) | 1 (4.8%) | 6 (28.6%) | 4 (19.0%) | 3 (14.3%) | 2 (9.5%) | 2 (9.5%) | 4 (19.0%) | 6 (28.6%) | 0 (0.0%) | 2 (9.5%) | 5 (23.8%) | 3 (14.3%) | 5 (23.8%) | 9 (42.9%) | 8 (38.1%) | 6 (28.6%) | 12 (57.1%) | 8 (38.1%) | 14 (66.7%) |
|  |  | Rs | -0.6 | 0.6 | -0.7 | -0.3 | 1.0 | 0.1 | -0.2 | -0.1 | -0.2 | -1.0 | 0.4 | -0.2 | -0.6 | -1.0 | 0.4 | -0.3 | -0.2 | 0.6 | 2.6 | -0.4 |
|  |  | **1-2 t/w** | 3 (14.3%) | 2 (9.5%) | 7 (33.3%) | 10 (47.6%) | 10 (47.6%) | 6 (28.6%) | 8 (38.1%) | 5 (23.8%) | 5 (23.8%) | 5 (23.8%) | 2 (9.5%) | 10 (47.6%) | 4 (19.0%) | 7 (33.3%) | 5 (23.8%) | 6 (28.6%) | 10 (47.6%) | 8 (38.1%) | 6 (28.6%) | 4 (19.0%) |
|  |  | Rs | 0.4 | -1.2 | 0.2 | -0.1 | 0.9 | -0.2 | -1.2 | -0.1 | -1.1 | 0.6 | -1.4 | -1.3 | -2.0 | 0.2 | 0.6 | 0.2 | 1.7 | 0.2 | 0.4 | -0.1 |
|  |  | **3-4 t/w** | 6 (28.6%) | 5 (23.8%) | 3 (14.3%) | 3 (14.3%) | 6 (28.6%) | 8 (38.1%) | 4 (19.0%) | 6 (28.6%) | 5 (23.8%) | 9 (42.9%) | 12 (57.1%) | 4 (19.0%) | 13 (61.9%) | 1 (4.8%) | 5 (23.8%) | 2 (9.5%) | 2 (9.5%) | 0 (0.0%) | 0 (0.0%) | 0 (0.0%) |
|  |  | Rs | -0.2 | -0.6 | -0.4 | -0.9 | -1.2 | -0.5 | -0.5 | 0.4 | 1.1 | -0.1 | 1.8 | 1.2 | 2.2 | -1.9 | 0.1 | -0.4 | -2.2 | -1.6 | -2.3 | -0.7 |
|  |  | **5-6 t/w** | 3 (14.3%) | 6 (28.6%) | 3 (14.3%) | 3 (14.3%) | 2 (9.5%) | 4 (19.0%) | 5 (23.8%) | 2 (9.5%) | 4 (19.0%) | 5 (23.8%) | 4 (19.0%) | 2 (9.5%) | 1 (4.8%) | 5 (23.8%) | 2 (9.5%) | 3 (14.3%) | 3 (14.3%) | 1 (4.8%) | 6 (28.6%) | 2 (9.5%) |
|  |  | Rs | 0.1 | 1.2 | 1.0 | 1.9 | -0.2 | 0.9 | 2.0 | -0.9 | 0.6 | -0.4 | -0.7 | 2.1 | 0.6 | 2.4 | -0.9 | 0.7 | 1.9 | 0.1 | 1.0 | 2.1 |
|  |  | **1-2 t/d** | 6 (28.6%) | 6 (28.6%) | 2 (9.5%) | 1 (4.8%) | 0 (0.0%) | 1 (4.8%) | 2 (9.5%) | 4 (19.0%) | 1 (4.8%) | 2 (9.5%) | 1 (4.8%) | 0 (0.0%) | 0 (0.0%) | 3 (14.3%) | 0 (0.0%) | 2 (9.5%) | 0 (0.0%) | 0 (0.0%) | 1 (4.8%) | 1 (4.8%) |
|  |  | Rs | 0.0 | 0.2 | 0.4 | 0.6 | -0.7 | 0.1 | 0.8 | 0.9 | 0.1 | 0.8 | -0.6 | 0.0 | 0.0 | 1.9 | -1.0 | 0.8 | -0.7 | 0.0 | -1.8 | 0.1 |
|  |  | **>3 t/d** | 2 (9.5%) | 1 (4.8%) | 0 (0.0%) | 0 (0.0%) | 0 (0.0%) | 0 (0.0%) | 0 (0.0%) | 0 (0.0%) | 0 (0.0%) | 0 (0.0%) | 0 (0.0%) | 0 (0.0%) | 0 (0.0%) | 0 (0.0%) | 0 (0.0%) | 0 (0.0%) | 0 (0.0%) | 0 (0.0%) | 0 (0.0%) | 0 (0.0%) |
|  |  | Rs | 0.4 | 0.6 | 0.0 | 0.0 | 0.0 | 0.0 | 0.0 | -0.7 | 0.0 | 0.0 | 0.0 | 0.0 | 0.0 | -0.7 | 0.0 | -1.0 | -0.7 | 0.0 | -0.7 | -0.7 |
|  | **Statistical values** | **X^2^** | 13.563 | 12.489 | 10.021 | 10.059 | 12.239 | 9.208 | 15.994 | 11.945 | 14.622 | 17.467 | 6.405 | 6.469 | 11.760 | 18.719 | 13.603 | 9.408 | 20.667 | 5.572 | 27.869 | 11.808 |
|  |  | **p** | 0.559 | 0.642 | 0.614 | 0.611 | 0.427 | 0.685 | 0.192 | 0.683 | 0.263 | 0.133 | 0.894 | 0.692 | 0.227 | 0.227 | 0.327 | 0.855 | 0.148 | 0.782 | 0.022 | 0.693 |
|  |  | **V** | 0.262 | 0.251 | 0.225 | 0.225 | 0.249 | 0.216 | 0.284 | 0.246 | 0.272 | 0.297 | 0.180 | 0.181 | 0.244 | 0.307 | 0.262 | 0.218 | 0.323 | 0.168 | 0.375 | 0.244 |

spec.: specific; t/w: times per week; Rs: residual; t/d: times per day; χ²: chi-square test value; V: Cramer’s V; prep/froz: prepared/frozen foods.

Supplementary Table S3 shows the distribution of adherence to dietary recommendations among athletes, considering the full sample. Athletes were below the dietary recommendations for fruits (92.4%), vegetables (97.0%), legumes (71.2%), cereals (86.4%), fish (84.8%), nuts (63.6%), and water intake (98.5%). With respect to red meat, eggs, and dairy products, athletes met the dietary recommendations (54.5%, 40.9%, and 68.2%, respectively). Finally, the only dietary recommendation that athletes exceeded was for meat and meat products (77.3%).

Table S3. Distribution (%) of adherence to dietary recommendations among athletes, total sample.

| Recommendations | **Total (n = 66)** | | |
| --- | --- | --- | --- |
|  | **Meets** | **Below** | **Above** |
| Fruits | 5 (7.6%) | 61 (92.4%) | - |
| Vegetables | 2 (3.0%) | 64 (97.0%) | - |
| Legumes | 14 (21.2%) | 47 (71.2%) | 5 (7.6%) |
| Cereals | 9 (13.6%) | 57 (86.4%) | - |
| Meat and meat products | 10 (15.2%) | 5 (7.6%) | 51 (77.3%) |
| Red meat | 36 (54.5%) | - | 30 (45.5%) |
| Eggs | 27 (40.9%) | 18 (27.3%) | 21 (31.8%) |
| Fish | 10 (15.2%) | 56 (84.8%) | - |
| Nuts | 19 (28.8%) | 42 (63.6%) | 5 (7.6%) |
| Dairy products | 45 (68.2%) | 21 (31.8%) | - |
| Water | 1 (1.5%) | 65 (98.5%) | - |

–: no recommendation available.

Supplementary Table S4 shows the distribution of adherence to dietary recommendations according to functional classification. No significant differences were observed in any of the dietary recommendations analysed (p = 0.109–0.705).

Table S4. Distribution (%) of adherence to dietary recommendations according to functional classification.

| Recomendations | **Functional classification** | | | | | | | | | | | | | | |
| --- | --- | --- | --- | --- | --- | --- | --- | --- | --- | --- | --- | --- | --- | --- | --- |
|  | **Gr 1 (n = 14)** | | | **Gr 2 (n = 10)** | | | **Gr 3 (n = 21)** | | | **Gr 4 (n = 21)** | | | **Statistical values** | | |
|  | **Meets** | **Below** | **Above** | **Meets** | **Below** | **Above** | **Meets** | **Below** | **Above** | **Meets** | **Below** | **Above** | **X^2^** | ***p*** | **V** |
| Fruits | 0 (0.0%) | 14 (100.0%) | - | 1 (10.0%) | 9 (90.0%) | - | 2 (9.5%) | 19 (90.5%) | - | 2 (9.5%) | 19 (90.5%) | - | 1.459 | 0.692 | 0.149 |
| *Residual* | *-1.2* | *1.2* |  | *0.3* | *-0.3* |  | *0.4* | *-0.4* |  | *0.4* | *-0.4* |  |  |  |  |
| Vegetables | 0 (0.0%) | 14 (100.0%) | - | 1 (10.0%) | 9 (90.0%) | - | 0 (0.0%) | 21 (100.0%) | - | 1 (4.8%) | 20 (95.2%) | - | 2.961 | 0.398 | 0.212 |
| *Residual* | *-0.7* | *0.7* |  | *1.4* | *-1.4* |  | *-1.0* | *1.0* |  | *0.6* | *-0.6* |  |  |  |  |
| Legumes | 3 (21.4%) | 11 (78.6%) | 0 (0.0%) | 2 (20.0) | 7 (70.0%) | 1 (10%) | 6 (28.6%) | 14 (66.7%) | 1 (4.8%) | 3 (14.3%) | 15 (71.4%) | 3 (14.3%) | 3.794 | 0.705 | 0.170 |
| *Residual* | *0.0* | *0.7* | *-1.2* | *-0.1* | *-0.1* | *0.3* | *1.0* | *-0.6* | *-0.6* | *-0.9* | *0.0* | *1.4* |  |  |  |
| Grains | 1 (7.1%) | 13 (92.9%) | - | 2 (20.0%) | 8 (80.0%) | - | 2 (9.5%) | 19 (90.5%) | - | 4 (19.0%) | 17 (81.0%) | - | 1.669 | 0.644 | 0.159 |
| *Residual* | *-0.8* | *0.8* |  | *0.6* | *-0.6* |  | *-0.7* | *0.7* |  | *0.9* | *-0.9* |  |  |  |  |
| Meat and meat products | 4 (28.6%) | 1 (7.1%) | 9 (64.3%) | 0 (0.0%) | 1 (10.0%) | 9 (90%) | 4 (19.0%) | 2 (9.5%) | 15 (71.4%) | 2 (9.5%) | 1 (4.8%) | 18 (85.7%) | 5.036 | 0.539 | 0.195 |
| *Residual* | *1.6* | *-0.1* | *-1.3* | *-1.5* | *0.3* | *1.0* | *0.6* | *0.4* | *-0.8* | *-0.9* | *-0.6* | *1.1* |  |  |  |
| Red meat | 8 (57.1%) | - | 6 (42.9%) | 7 (70.0%) | - | 3 (30.0%) | 14 (66.7%) | - | 7 (33.3%) | 7 (33.3%) | - | 14 (66.7%) | 6.057 | 0.109 | 0.303 |
| *Residual* | *0.2* |  | *-0.2* | *1.1* |  | *-1.1* | *1.4* |  | *-1.4* | *-2.4* |  | *2.4* |  |  |  |
| Eggs | 5 (35.7%) | 4 (28.6%) | 5 (35.7%) | 4 (40.0%) | 3 (30.0%) | 3 (30.0%) | 6 (28.6%) | 7 (33.3%) | 8 (38.1%) | 12 (57.1%) | 4 (19.0%) | 5 (23.8%) | 3.829 | 0.700 | 0.170 |
| *Residual* | *-0.4* | *0.1* | *0.4* | *-0.1* | *0.2* | *-0.1* | *-1.4* | *0.8* | *0.7* | *1.8* | *-1.0* | *-1.0* |  |  |  |
| Fish | 1 (7.1%) | 13 (92.9%) | - | 1 (10%) | 9 (90.0%) | - | 2 (9.5%) | 19 (90.5%) | - | 6 (28.6%) | 15 (71.4%) | - | 4.364 | 0.225 | 0.257 |
| *Residual* | *-0.9* | *0.9* |  | *-0.5* | *0.5* |  | *-0.9* | *0.9* |  | *2.1* | *-2.1* |  |  |  |  |
| Nuts | 4 (28.6%) | 10 (71.4%) | 0 (0.0%) | 2 (20.0%) | 8 (80.0%) | 0 (0.0%) | 7 (33.3%) | 12 (57.1%) | 2 (9.5%) | 6 (28.6%) | 12 (57.1%) | 3 (14.3%) | 4.424 | 0.620 | 0.183 |
| *Residual* | *0.0* | *0.7* | *-1.2* | *-0.7* | *1.2* | *-1.0* | *0.6* | *-0.7* | *0.4* | *0.0* | *-0.7* | *1.4* |  |  |  |
| Dairy products | 9 (64.3%) | 5 (35.7%) | - | 8 (80.0%) | 2 (20.0%) | - | 11 (52.4%) | 10 (47.6%) | - | 17 (81.0%) | 4 (19.0%) | - | 4.737 | 0.192 | 0.268 |
| *Residual* | *-0.4* | *0.4* |  | *0.9* | *-0.9* |  | *-1.9* | *1.9* |  | *1.5* | *-1.5* |  |  |  |  |
| Water | 1 (7.1%) | 13 (92.9%) | - | 0 (0.0%) | 10 (100.0%) | - | 0 (0.0%) | 21 (100.0%) | - | 0 (0.0%) | 21 (100.0%) | - | 3.771 | 0.287 | 0.239 |
| *Residual* | *1.9* | *-1.9* |  | *-0.4* | *0.4* |  | *-0.7* | *0.7* |  | *-0.7* | *0.7* |  |  |  |  |

–: no recommendation available; χ²: chi-square test value; V: Cramer’s

Supplementary Table S5 shows the distribution of responses related to water consumption throughout the day, during training sessions, and during matches, according to functional classification. The only significant difference found was in water consumption during matches (p = 0.041), with athletes classified as grade 1 showing proportionally lower consumption and those classified as grade 4 showing proportionally higher consumption. No significant differences were observed for water consumption during the day or during training sessions (p = 0.524 and 0.451, respectively).

Table S5. Distribution (%) of responses regarding water intake according to functional classification.

| Questions | Response options | **Functional classification** | | | | | | | | | | |
| --- | --- | --- | --- | --- | --- | --- | --- | --- | --- | --- | --- | --- |
|  |  | **Gr 1 (n = 14)** | ***Rs*** | **Gr 2 (n = 10)** | ***Rs*** | **Gr 3 (n = 21)** | ***Rs*** | **Gr 4 (n = 21)** | ***Rs*** | **Statistical values** | | |
|  |  |  |  |  |  |  |  |  |  | **X^2^** | ***p*** | **V** |
| Liters per day | 0.5 L | 1 (7.1%) | *1.0* | 0 (0.0%) | *-0.6* | 1 (4.8%) | *0.6* | 0 (0.0%) | *-1.0* | 11.062 | 0.524 | 0.236 |
|  | 1 L | 3 (21.4%) | *0.7* | 2 (20.0%) | *0.5* | 4 (19.0%) | *0.6* | 1 (4.8%) | *-1.6* |  |  |  |
|  | 1.5 L | 1 (7.1%) | *-0.8* | 0 (0.0%) | *-1.4* | 5 (23.8%) | *1.6* | 3 (14.3%) | *0.1* |  |  |  |
|  | 2 L | 5 (35.7%) | *0.8* | 4 (40.0%) | *1.0* | 3 (14.3%) | *-1.6* | 6 (28.6%) | *0.2* |  |  |  |
|  | 2.5 L | 4 (28.6%) | *-1.1* | 4 (40.0%) | *-0.1* | 8 (38.1%) | *-0.3* | 11 (52.4%) | *1.3* |  |  |  |
| Litres of water consumed during training sessions | 0.5 L | 6 (42.9%) | *2.0* | 3 (30.0%) | *0.6* | 4 (19.0%) | *-0.5* | 2 (9.5%) | *-1.7* | 11.928 | 0.451 | 0.245 |
|  | 1 L | 5 (35.7%) | *-0.6* | 5 (50.0%) | *0.5* | 8 (38.1%) | *-0.5* | 10 (47.6%) | *0.6* |  |  |  |
|  | 1.5 L | 3 (21.4%) | *-0.1* | 1 (10.0%) | *-1.0* | 4 (19.0%) | *-0.5* | 7 (33.3%) | *1.4* |  |  |  |
|  | 2 L | 0 (0.0%) | *-1.5* | 1 (10.0%) | *-0.1* | 4 (19.0%) | *1.5* | 2 (9.5%) | *-0.2* |  |  |  |
|  | Does not usually drink wate | 0 (0.0%) | *-0.5* | 0 (0.0%) | *-0.4* | 1 (4.8%) | *1.5* | 0 (0.0%) | *-0.7* |  |  |  |
| Liters of water consumed during matches | 0.5 L | 8 (57.1%) | *3.0* | 2 (20.0%) | *-0.5* | 5 (23.8%) | *-0.2* | 2 (9.5%) | *-2.1* | 21.723 | 0.041 | 0.331 |
|  | 1 L | 6 (42.9%) | *1.0* | 3 (30.0%) | *-0.1* | 7 (33.3%) | *0.1* | 5 (23.8%) | *-1.0* |  |  |  |
|  | 1.5 L | 0 (0.0%) | *-2.4* | 4 (40.0%) | *1.3* | 5 (23.8%) | *-0.1* | 7 (33.3%) | *1.2* |  |  |  |
|  | 2 L | 0 (0.0%) | *-1.7* | 1 (10.0%) | *-0.4* | 2 (9.5%) | *-0.7* | 6 (28.6%) | *2.4* |  |  |  |
|  | 2.5 L | 0 (0.0%) | *-0.9* | 0 (0.0%) | *-0.7* | 2 (9.5%) | *1.3* | 1 (4.8%) | *0.1* |  |  |  |

Rs: residual; L: liters χ²: chi-square test value; V: Cramer’s V
